# Perceptions of sedentary behaviour in people with severe asthma: a qualitative study

**DOI:** 10.1186/s12889-024-20446-4

**Published:** 2024-10-30

**Authors:** Paola D Urroz Guerrero, Peter G Gibson, Hayley Lewthwaite, Eleanor Majellano, Sarah A Hiles, Vanessa M McDonald

**Affiliations:** 1 Centre for Research Excellence in Treatable Traits, College of Health, Medicine, and Wellbeing, University of Newcastle, New Lambton Heights, NSW Australia; 2 Asthma and Breathing Program, Hunter Medical Research Institute, Level 2 West Locked Bag 1000, New Lambton Heights, NSW Australia; 3 Department of Respiratory and Sleep Medicine, John Hunter Hospital, New Lambton Heights, NSW Australia; 4 School of Psychological Sciences, University of Newcastle, Callaghan, NSW Australia

**Keywords:** Severe asthma, Sedentary behaviour, Qualitative

## Abstract

People with severe asthma often lead sedentary lifestyles, which adversely affects overall health and asthma-specific outcomes. To inform future sedentary behaviour- interventions, this study aimed to explore perceptions of sedentary behaviour among people with severe asthma. Adults (≥ 18 years) with severe asthma (*n* = 21) participated in face-to-face interviews. Participants were asked open-ended questions about factors influencing their sitting behaviour. A thematic analysis was conducted on phrases and sentences relevant to sedentary behaviour. Participants were predominantly females (62%), with controlled asthma (median [Q1, Q3]: ACQ6 0.5 [0.2,1.8]) and receiving monoclonal antibody therapy (71%). Almost half of the participants were not meeting the physical activity guidelines (47%) and were sedentary (10.8 [9.7, 11.4] hours of sedentary behaviour per day). The analysis generated four main themes: (1) Sedentary behaviour often stems from habits and routines, (2) Asthma and associated health issues contribute to sedentary behaviour, (3) Participants’ responsibilities influence their activity levels, and; (4) Participants’ conscious balance between being physically active and sedentary. The results of this qualitative study offers insights into the perspectives of people with severe asthma regarding sedentary behaviour, highlighting the identification of strategies that can be implemented to improve sedentary behaviour in this population.

**Supplementary Information:**

The online version contains supplementary material available at 10.1186/s12889-024-20446-4.

## Introduction

Sedentary behaviour is associated with an increased risk of cardiovascular disease, diabetes, and all-cause mortality [1]. There is evidence that the negative health consequences of prolonged sedentary behaviour are independent of moderate to vigorous physical activity (MVPA) levels [2]. For people with asthma, more time spent being sedentary is additionally associated with decreased exercise capacity, lung function, and asthma control [3]. Despite the deleterious effects of sedentary behaviour on important health and asthma-specific outcomes, people with severe asthma spend up to nine hours per day being sedentary, with half of this time spent in sitting bouts longer than 30 min [4]. Targeting sedentary behaviour as part of asthma management warrants investigation as this approach may positively impact people with severe asthma.

In 2020, for the first time, the World Health Organisation physical activity guidelines included recommendations for limiting the amount of time spent sedentary across all age groups and abilities [5]. The inclusion of sedentary behaviour recommendations in guidelines is important, as inactivity and sedentariness are separate constructs. Inactive refers to not meeting the recommended amount of MVPA. Sedentary refers to spending a high proportion of wake time in sedentary activities; any waking behaviour characterised by energy expenditure ≤ 1.5 metabolic equivalents (METs), while in a sitting, reclining or lying posture [6]. It is therefore possible to be active and sedentary, or inactive yet not sedentary. For example, an individual can be considered active yet sedentary if they spend most of their day sitting at work (sedentary) but regularly engage in exercise outside of work (active). Conversely, an individual may be inactive yet not sedentary if they spend the majority of their day performing light household tasks (non-sedentary) without participating in physical activities of at least moderate intensity (inactive). Consequently, addressing inactivity is different to addressing sedentariness [7].

There is limited research addressing how to address sedentary behaviour in people with severe asthma [8]. Existing randomised controlled trials (RCT) have primarily focused on the impact of physical activity interventions on both activity and sedentary behaviour outcomes [9–11]. To date, there are no RCT that have implemented a sedentary behaviour focused intervention in people with severe asthma. Interventions targeting sedentary behaviour in the general population and in chronic disease conditions have shown promising results when solely focusing on sedentary behaviour and not co-targeting other health behaviours like physical activity and diet [7].

Behaviour change interventions are complex, requiring a systematic approach to understand how and under what circumstances change in behaviour is achieved [12, 13]. The United Kingdom Medical Research Council’s framework for the development of complex interventions identifies the importance of involving key stakeholders to develop programme theory [13]. Taking this into consideration, the perspectives of people with severe asthma are needed to inform a sedentary behaviour targeted intervention; however, this is yet to be investigated. To address this knowledge gap, we sought to explore perceptions of sedentary behaviour among people with severe asthma.

## Methods

### Study design

A descriptive qualitative study was conducted as part of a single centre, parallel group RCT in people with severe asthma, reported previously [14]. Briefly, participants were randomised to a yoga and mindfulness intervention or usual care with tailored information about physical activity, mindfulness, and goal setting. After completing the trial, all participants were interviewed face-to-face regarding perceptions of physical activity, sedentary behaviour and perceived benefits and weaknesses of the intervention.

Ethics approval was obtained from the Hunter New England Human Research Ethics Committee (2018/ETH00338) and the trial was registered on the Australia New Zealand Clinical Trials Register (ACTRN12618001914257). Written informed consent was obtained from each participant prior to data collection in accordance with Good Clinical Practice. This study is reported according to the Consolidated Criteria for REporting Qualitative (COREQ) [15].

### Participant recruitment and selection

Adults (≥ 18 years) with severe asthma (defined according to the European Respiratory Society (ERS) / American Thoracic Society (ATS) Task force definition [16]) were recruited to the RCT. Details of inclusion and exclusion criteria have been previously reported [14]. Participants were recruited *via* consecutive methods through the research database and clinics of the Department of Respiratory and Sleep Medicine at John Hunter Hospital, NSW and via general/social media advertisement. Expressions of interest were extended via treating respiratory physicians, who referred eligible patients to the study. A total of 24 participants recruited and randomised to the RCT. All participants were invited to a face-to-face semi structured interview within 2 months of completing the intervention on an intention to treat basis [14].

### Assessment

The assessment included collecting self-reported demographic information, asthma and exacerbation history (previous 12 months), and respiratory medication use. Clinical measures were also completed by participants as part of their participation in the RCT [14].

Pre and post-bronchodilator (400 µg of Salbutamol) spirometry according to ATS/ERS guidelines was completed to attain measures of lung function and 2012 GLI equations were used to calculate predicted values [17]. Participant’s functional exercise capacity was measured using the 6-minute walk test (6MWT), to calculate 6-minute walk distance (6MWD), according to guidelines [18]. Participants wore a tri-axial accelerometer (Actigraph wGT3X-BT) for eight consecutive days to measure moderate-to-vigorous physical activity (MVPA), light-intensity physical activity (LPA) and sedentary time. Tri-axial accelerometer data processing details have been described previously [19]. The Asthma Control Questionnaire (ACQ) was administered to assess the level of asthma control [20]. Health-related quality of life was measured using the St. George’s Respiratory Questionnaire (SGRQ) [21]. The Dyspnoea-12 (D-12) questionnaire was completed by participants to assess breathlessness [22]. Levels of anxiety and depression symptoms were measured using the Hospital Anxiety and Depression Scale (HADS) [23].

### Interview procedure

The interviews took place in a private room at the Hunter Medical Research Centre (Newcastle, NSW, Australia) for a mean (SD) duration of 97 min (21). Present during the interviews were the participant and the facilitator. A semi-structured, open-ended interview guide was used. Participants were asked open-ended questions from an interview guide that explored their perspectives on physical activity, sedentary behaviour, and the perceived benefits and limitations of the intervention. The interview guide included specific questions aimed at understanding factors influencing participants’ sitting behaviour (see Supplementary material). For this study, we conducted a secondary analysis focusing on responses related to sedentary behaviour, including both direct answers to sedentary behaviour questions and relevant phrases and sentences made throughout the interview. The facilitator was author SAH (PhD), a female post-doctoral researcher who is trained in qualitative research, did not have an established role in providing clinical care to the participants and did not deliver the intervention as part of the RCT.

### Data generation and analysis

Descriptive statistics were calculated using SPSS version 28 (IBM, Armonk, NY, USA). The interviews were audio-recorded and transcribed verbatim. Participants were informed they may review and edit their transcript of the interview, one participant reviewed and edited their transcript. Transcripts were pseudonymised to protect confidentiality. Data familiarisation, both in written form and audio recording was undertaken [24, 25]. The analysis accounted for field notes, vocal tone, and participant emphasis on certain words and phrases to enable underlying emotions to be understood within the context of the conversation.

Inductive thematic analysis was undertaken according to Braun and Clarke’s six steps approach [25] by author PUG using NVivo 12 Pro (QSR International, Melbourne, VIC, Australia). Data were reviewed line by line to capture relevant data to sedentary behaviour. As a concept became apparent codes assigned, organising the data into meaningful groups. Themes were then generated from the codes. Saturation was achieved when the identified themes demonstrated robustness, with no novel information emergent from the data. A second qualitative researcher (EM) independently coded 30% of the transcripts, with comparison made to resolve discrepancies. Coding agreement was achieved with a mean (SD) percentage agreement of 99.9 (0.3). Generated codes and themes, thematic saturation and the interpretation of the findings were discussed and reviewed regularly with co-authors PG, HL and VM.

## Results

### Participant characteristics

A total of 21 participants attended and completed the face-to-face semi structured interview [14]. All 21 interview transcripts were included in the analysis, 62% of which were participants who were randomised to the yoga and mindfulness intervention and the remaining in the active control group [14]. Participant’s demographics, clinical and movement characteristics are summarised in Table 1. Participants were mostly females (62%) with a mean (SD) body mass index (BMI) of 31.9 (5.9) kg/m^2^. Their asthma was controlled with a median (quartile 1; quartile 3) ACQ of 0.5 (0.2; 1.8), and most participants were receiving monoclonal antibody therapy for severe asthma (71%). (Table 1). Participants participated in a median (quartile 1; quartile 3) of 19.1 (11.3; 44.5) minutes of MVPA and 10.8 (9.7; 11.4) hours of sedentary time per day.

**Table 1:** Participant demographic and asthma-related characteristics

Demographic characteristics	*n* = 21
Sex, females (n, %)	13 (62)
Age, years (mean ± SD)	67 ± 9
BMI, kg/m^2^ (mean ± SD)	31.9 ± 5.9
Age of diagnosis, years (median, Q1; Q3)	33 (5; 49)
Race (n, %)	
Caucasian	20 (95%)
Aboriginal or Torres Strait Islander	1 (5%)
Living arrangement* (n, %)	
Living alone	5 (24)
Living with spouse/partner	12 (57)
Living with family	3 (14)
Employment or study status (n, %)	
Working fulltime or part time	7 (33)
Not working or retired	14 (67)
**Medications**	
Prescribed monoclonal antibody therapy (n, %)	15 (71%)
Beclomethasone daily equivalent, mcg (mean ± SD)	1771 ± 811
**Clinical measures**	
ACQ6, score (median, Q1; Q3)	0.5 (0.2; 1.8)
Post BD FEV_1_, % predicted (mean ± SD)	77.1 ± 20.8
Post BD FEV_1_/FVC, ratio (mean ± SD)	0.67 ± 0.14
6MWD, m (mean ± SD)	486.8 ± 115.4
**Self-reported measures**	
SGRQ, total score (mean ± SD)	36.8 ± 20.9
D12, total score (mean ± SD)	10.0 ± 8.8
HADS-Anxiety, sub score (mean ± SD)	5.4 ± 3.7
HADS-Depression, sub score (mean ± SD)	4.4 ± 4.0
HADS, total score (mean ± SD)	9.8 ± 6.9
Exacerbation History (previous 12 months)	
Hospital admissions (n, %)	3 (14)
Emergency department admissions (n, %)	4 (19)
General practitioner visits (n, %)	6 (29)
OCS use (n, %)	17 (81)
Antibiotic use (n, %)	15 (71)
Total exacerbation rate	2 (1, 4)
Movement Behaviours (per day)**	
Not meeting physical activity guidelines (n, %)	8 (47)
Moderate to vigorous physical activity, minutes (median, Q1; Q3)	19.1 (11.3; 44.5)
Light intensity physical activity, hours (median, Q1; Q3)	2.3 (1.8; 3.0)
Sedentary time, hours (median, Q1; Q3)	10.8 (9.7; 11.4)

6MWD, 6-minute walk distance; ACQ, Asthma control questionnaire; BD, Bronchodilator; BMI, Body mass index; D12, Dyspnoea-12; FEV_1_, Forced expiratory volume in 1 s; FVC, Forced vital capacity; HADS, Hospital anxiety and depression scale; OCS, Oral corticosteroids; SGRQ, St. George respiratory questionnaire. *missing data from *n* = 1 **missing data from *n* = 4

### Themes

**Fig. 1:**
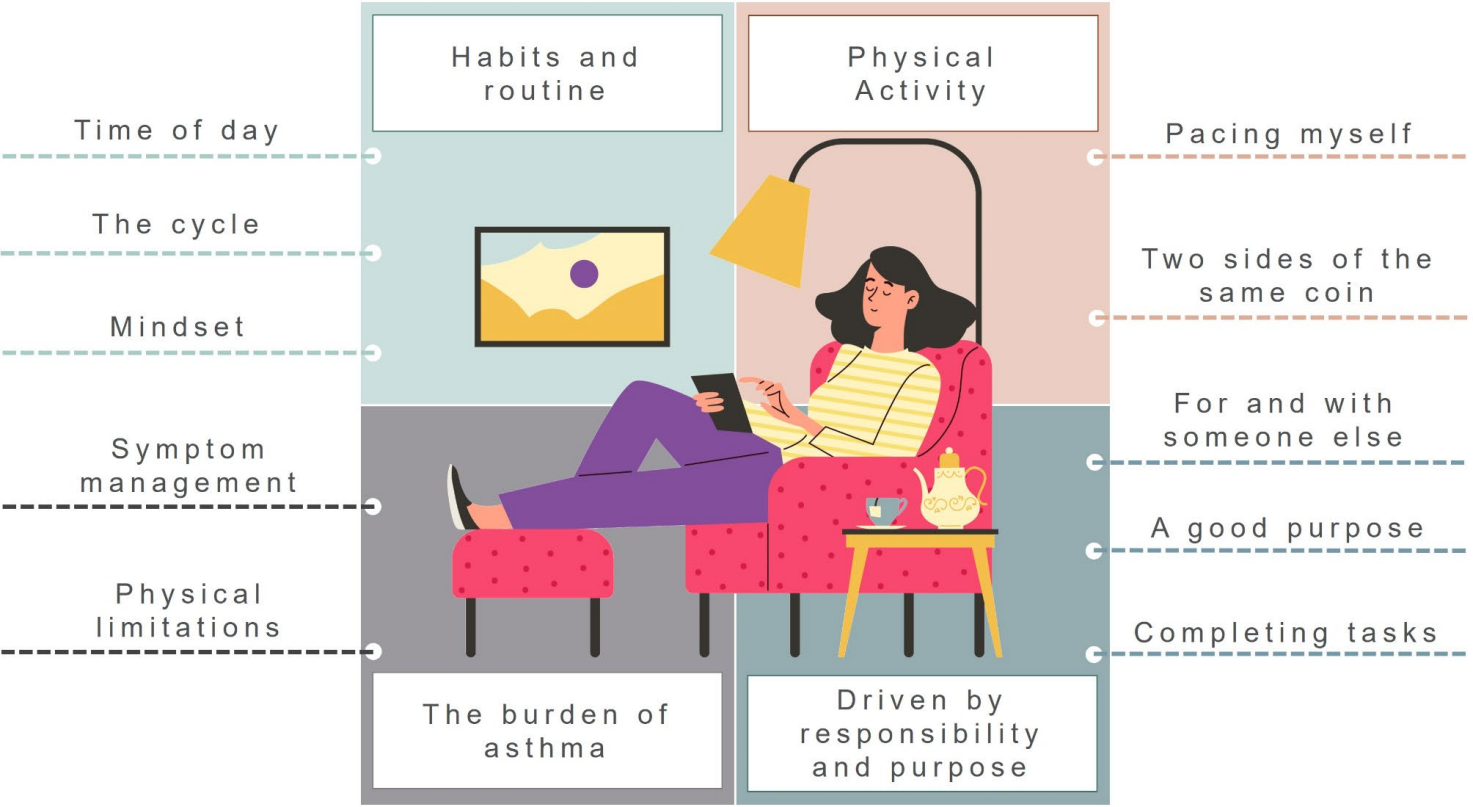
Conceptual diagram of generated themes and subthemes

During the thematic analysis process, data saturation was achieved with 20 participants (no new information emerged from the data) and 14 codes were reviewed and collated into themes. The analysis generated four main themes and 10 subthemes (Fig. 1; Table 2).

**Table 2:** Exemplar quotes for themes and subthemes

Theme 1: Habits and Routine
*Subtheme: Time of day*
“Yeah, I’d prefer to do something morning rather than anything in the afternoon. Mainly because I think by the afternoon, if I do have a busy morning, the afternoons - it’s just rest time” - Female, 64 years “Um so, I’ve always been like someone who’s got 10 things to do that day, so I’ll get them all done and have my like little job list and then I’ll sit down of a night” - Female, 49 years “I’m a morning person, so I tend to be a little bit more active in the morning and then in the afternoon, after lunch, is when I normally do my sewing or do my Scrabble or doing things like I just sit down” - Female, 57 years “… Yeah, in the evening where I’ll sit down and maybe for four hours, four or five hours in the evening sitting there, working - looking at the computer and TV and such. During the daytime, I try to keep moving around doing little things around the home and in the backyard; or I have a friend who I sometimes go out with” - Male, 68 years
*Subtheme: The cycle*
“Sometimes, I can’t even stand the ads and I say, there is a break, get out of it. If you watch that again, you will go totally mad. So you get up and you move” - Female, 77 years “It’s not - it makes me lazy; it does. If I sit around for too long then I really don’t want to get up and do anything” - Female, 64 years “It sounds like a bit of a cycle. You don’t want to get up so you don’t get up and then that makes you less likely to want to get up” - Female, 64 years “…you have to be wary of just sitting there watching and that’s where I find this getting up and doing something in between and even if it’s just getting out of the chair, walking out and wiping the dishes, making a cup of tea, looking around in your kitchen and say oh, I’ll put that there for tomorrow or whatever and not just sit totally for a long period” - Female, 77 years
*Subtheme: Mindset*
“So it’s not just your lungs, it’s your bones and your blood circulation. So if you don’t keep all those going, the whole thing is just - it’s like a car that sits in the garage; it won’t start after a while, you know?” - Female, 77 years “The other bit is getting on with life, people who sit too much don’t do enough, I don’t think” - Male, 63 years “You’ve got to keep moving, get up and - even if you’re in the house in a dressing gown, move because that’s wrong” - Female, 77 years “Sometimes I don’t feel - like, I think, yeah, maybe I’ll go out today and then I think, no, I think I’ll just stay at home, where I probably should push myself to get dressed and go out, but I don’t” - Female, 64 years
**Theme 2: The burden of asthma**
*Subtheme: Symptom management*
“I spent two whole weeks in bed, I didn’t move. So, 1000 steps in a day was huge for me because I was so sick. Getting up and going to the toilet and then getting back into bed was about as far as I went…” - Female, 49 years “That’s all you think about is moving and breathing, moving and breathing, and it’s slow moving and it’s not puffing you out and it’s not making your asthma any worse. Then you can actually sit down and go I can actually breathe better. As soon as you start to breathe a little bit better, your mind is going I’m good, I’m okay now, and the clouds clear” - Female, 49 years “If I’m not feeling well, if my cough is bad, after dinner every night, I’ll park myself in front of the TV and sit there and cough and carry on and watch TV until the early hours of the morning” - Male, 73 years “…if I’m having an asthma attack, yeah and I sit down to relieve that” - Female, 62 years
*Subtheme Physical limitations*
“I’d just like to be able to do things around the house without any pain and without - to be without pain would be unbelievable” - Female, 64 years “I don’t get up in a hurry. I sit up and then I’ve usually got to push myself out of bed these days because I’m not as strong in the knees, and I put that down to lack of exercise” - Male, 63 years “So that’s when I’ll sit down and read because I won’t go to sleep because I’ll be a bit lethargic and think, oh, I’ve had enough today” - Female, 70 years “For me, if I open the door and it’s overcast and the wind is cold and it’s drizzly, I’d be better off to shut the door, go back inside and wait for a nice day…but if it’s sunny and warm outside, it - I’m more conducive to being outside doing things” - Male, 64 years
**Theme 3: Driven by responsibility and purpose**
*Subtheme: For and with someone else*
“His motivation has really fallen and I think that brings me down a bit, not having somebody that says, come on, let’s get up and go” - Female, 64 years “Probably if I’m on the phone to friends I’d be sitting yapping away” - Female, 61 years “I’m a sewer; I make quilts and take everybody’s clothes - the legs of pants takes in, darts here, the usual everyone piles it on and the family which I don’t mind” - Female, 77 years “So I’m sitting on my bum for four to six hours a day, whereas previously I wouldn’t be sitting on my backside for that period of time. But it’s something I want to do and something I’m doing that’s beneficial to somebody else. It’s not a paid position, but it’s something that I’m doing to help some friends out and I enjoy doing it. It’s keeping the mind active and that too, as well” - Male, 75 years
*Subtheme: A good purpose*
“…yesterday with the 3000 steps when I sat in front of the computer for three hours, you know doing something that that really fascinates me” - Male, 63 years “…so the sitting is a luxury, which I enjoy but it’s still a luxury and I’d like to always keep it that way” - Male, 63 years “I think that that then helps the rest of your day, it gives you the energy to be able to move on and do the important things. But you’ve got to make your body learn to relax again. Again, I suppose that’s where that sedentary part is actually an active sedentary part because it becomes important in the active parts of your day” - Male, 63 years “So I sit here and I relax my body and I’m good” - Male, 75 years
*Subtheme: Completing tasks*
“During the daytime, I try to keep moving around doing little things around the home and in the backyard” - Male, 68 years “I mean I do all the washing and cooking and cleaning up of the house. But I don’t do the floors or the bathrooms. I get someone to come in and help me with that. So, that takes a good, I suppose half an hour, hour a day. It depends what - this morning I’ve done a couple of loads of washing and just hung it out on the horse inside” - Female, 78 years “…and you want to just sit down in front of the couch, actually getting up to cook is another little activity that you do…” - Male, 64 years “If you’ve got a shopping day, and then you might decide that you’re going to do other things while you’re out shopping. So I won’t sit around much that day at all” - Female, 70 years
**Theme 4: Physical activity**
*Subtheme: Pacing myself*
“…and if I get up and do something active - and not all the time, because I think relax-, everybody needs to be able to relax, I think, that’s very important. You must smell the roses in your life” - Male, 63 years “I know it’s only going to be five or 10 min and I’m going to be running again. I’ve just run out of petrol. Just refuel and away you go” - Male, 75 years “When I do a really active day, the next day I’m not too active. Because I get quite tired” - Female, 64 years “I can rake for a little while but I can’t rake very long or push a broom very long because that runs me out of air. I can pick up rocks but I can’t pick up four rocks. I’ll only pick up one rock at a time for two rocks. Then I’ll go and sit down and then I’ll come back and I’ll pick up another two” - Male, 75 years
*Subtheme: Two sides of the same coin*
“Because I’m so active I’m not a particularly sedentary person” - Male, 63 years “…but being sedentary to me is part of your life but for me the more important the more enjoyable part of my life is doing the exercise” - Male, 63 years “Well, when I’m not feeling well, I just become inactive. It might be just a habit that I park myself in front of the TV and watch sport. I love watching sport on TV. I watch back - I watch old football games, old cricket games, go back 30 years and that sort of thing” - Male, 73 years “…because it’s like when you say less sedentary, it could be right, okay, look off you go and play tennis or something like that” - Female, 57 years

### Theme one: habits and routine

This theme describes how participants’ sedentary behaviour pattern is often a result of established habits and routine. The subtheme ‘time of day’ refers to participants’ formed habits and routine based on the time of the day, where the morning was dedicated to activities such as shopping, physical activity and social engagements and the evenings to being at home, sitting, and watching television. Another subtheme was ‘the cycle’ which represented participants’ habit of sitting for long periods uninterrupted and the conscious need to break this habit otherwise the cycle will continue. Activities that some participants described to break up their sedentary behaviour included household chores or gardening. However, participants indicated that their awareness of the need to break this habit did not always lead to acting on it. The third subtheme for habits and routine was ‘mindset’. This was formed to describe how participants’ self-perception or belief towards being a sedentary person influenced their established routines/habits to avoid sedentary behaviour.

*“I don’t want to be one of these old people that sit there waiting to die” - Female*,* 70 years*.

However, some participants described a variety of situations, such as an asthma exacerbation, where that mindset breaks and participants no longer had the mental ability to keep up their routines/habits to avoid being sedentary.

### Theme two: the burden of asthma

Participants highlighted that having asthma and associated multi-morbidity impacts the amount of sedentary behaviour they participate in. This further produced the subtheme ‘symptom management’ which extrapolated that their behaviour is dependent on how well their symptoms were managed. Participants particularly described how breathlessness is the most problematic symptom they experience and that they engage in sedentary behaviour to alleviate it.

*“I can sit down and concentrate and deep breathe and get out of the tight feeling and relax” - Female*,* 78 years*.

The subtheme ‘physical limitation’ captured the presence of physical limitations imposed by multimorbidity such as pain, fatigue, and how the physical environment such as cold and windy weather, reduces their capability and opportunities to participate in non-sedentary behaviour.

### Theme three: driven by responsibility and purpose

This theme described how participants’ roles or responsibilities dictate the type of activity they engage in, and consequently, if the activity is completed in a sedentary way or not. Within this theme is the subtheme ‘for or with someone else’. This refers to participants discussing how fulfilling a social or caring role contributes to the type of activity they engage in. One participant described that he would leave his “hermit stage” to be social with good friends. This subtheme also includes the impact that a fellow companion or partner has on their own behaviour, for example, a companion may encourage or discourage sedentary behaviour.

*“…my husband will come in and say*,* come on*,* we’re going to do something. You’ve been sitting there long enough” - Female*,* 70 years*.

Another formed subtheme is ‘a good purpose’. Participants described how they benefit from engaging in sedentary behaviour including time to reflect, relax, focus, and do hobbies. Lastly, the subtheme ‘completing tasks’ captured the responsibility or role participants held, particularly household tasks. These tasks lead to less sedentary behaviour as it fulfills their sense of purpose.

### Theme four: physical activity

Participants discussed the relationship between physical activity and sedentary behaviour. One subtheme for physical activity is ‘pacing myself’ which represents participants’ conscious balance between being physically active and sedentary. For example, if they pushed themselves beyond their physical capability, this led to prolonged sedentary behaviour. Whereas, if they paced themselves with their physical activity, their sedentary behaviour would be accumulated in shorter bouts.

*“For me to mow the grass*,* it might take me all day. But I get it done. I go and do one strip and come back. One strip and come back and sit down” - Male*,* 75 years*.

‘Two sides of the same coin’ is another subtheme that captures participants understanding about the difference between physical activity and sedentary behaviour. To the participants, being sedentary meant you are not doing enough physical activity and conversely, you are not being sedentary if you are doing enough physical activity.

*“…because it’s like when you say be less sedentary*,* it could be right*,* okay*,* look off you go and play tennis or something like that” - Female*,* 57 years*.

## Discussion

This qualitative study reports the perceptions of sedentary behaviour among people with severe asthma. There were three main findings from these data (1) while people with severe asthma perceived sedentary behaviour negatively when accumulated in long continuous bouts, they considered sedentary behaviour to be necessary to manage symptoms, rest, and relax; (2) sedentary behaviour patterns were part of established habits or routines, which were driven by the status of their asthma control, the roles and responsibilities they hold, and by external influences; and (3) we identified misconceptions in relation to physical inactivity and sedentary behaviour; participants often perceived that, to not be sedentary, meant you had to be doing enough MVPA. These data provide underpinning programme theory on sedentary behaviour in people with severe asthma, which should be considered when designing targeted sedentary behaviour interventions.

How people accumulate sedentary behaviour has different impacts on health outcomes. For example, two individuals may accumulate the same total sedentary time in a day, however, one might break up sedentary behaviour 30 times with bouts not exceeding 30 min, while the other might only have 10 breaks with sitting bouts frequently lasting longer than 30 min. Previous research shows that despite spending a similar amount of time being sedentary as non-asthma counterparts (9 h), people with severe asthma accumulate sedentary behaviour in longer bouts (> 30 min), which may be more deleterious for health (5 versus 4 h) [4]. In this study, people with severe asthma described their consciousness of participating in long periods of uninterrupted sedentary behaviour. Conversely, participants advocated for sedentary behaviour by describing the benefits of allocating time in their day to sit and participate in sedentary activities that are important to them. Additionally, sitting enabled the participants to engage in daily life activities, which is similar to a chronic obstructive pulmonary disease (COPD) population [26]. Therefore, sedentary behaviour is not necessarily always viewed as a negative behaviour for people with severe asthma. This finding has similarly been shown in an older adult population [27]. Specifically in our study, people with severe asthma commonly mentioned television watching as an activity that enables long periods of sedentary behaviour. Higher amounts of television-viewing is related to worse health outcomes irrespective of high levels of moderate intensity physical activity [28]. Additionally, participants in our study described the afternoon as being the most common time for long uninterrupted bouts of sitting. Therefore, the afternoon may be an opportunistic time of the day to break up sedentary time in people with severe asthma [29]. Interrupting these prolonged periods of sedentary behaviour with small bouts of physical activity (e.g. 2–5 min) may be a feasible option for people with severe asthma [30], however, this is yet to be investigated. Shared-decision making may also be required to find alternative types of behaviour that do not lead to prolonged sitting but continue to provide relaxation.

Symptoms such as breathlessness, pain, and fatigue impact the amount of sedentary behaviour that people with severe asthma engage in. When asthma symptoms were increased, participants described an inability to do anything requiring effort. This is similarly reported by people with rheumatoid arthritis, where the fluctuation of the inflammatory disease makes it a “constant battle” between days with low amounts of sedentary behaviour and days with high amounts of sedentary behaviour [31]. Having musculoskeletal conditions is common in people with severe asthma and is associated with spending a full day being sedentary [32, 33]. Specifically, the prevalence of osteoarthritis has been shown to be as high as 32% in people with asthma, a higher prevalence than in healthy controls and people with COPD [34]. Although we can not determine if osteoarthritis was the driver of pain causing sedentary behaviour in this study, people with asthma and people with osteoarthritis spend up to two thirds of their day being sedentary and attribute this to pain [35, 36]. These findings show the importance of managing asthma symptoms and comorbidities as part of a multi-dimensional approach to addressing sedentary behaviour in people with severe asthma. Further educating people with severe asthma about replacing sedentary behaviour with feasible options such as standing or LPA while experiencing symptoms may also be beneficial for their health and wellbeing.

People with severe asthma spoke about the importance of social roles and household responsibilities they hold. As with people with COPD [37], the behaviour of people with severe asthma is influenced by their family, friends, and household members. They expressed that their loved ones either facilitated sedentary behaviour, or conversely, active behaviours [37]. Individuals’ sedentary behaviour patterns have been shown to be linked to the behaviours of loved ones, such as partners or household members [38]. These findings warrant the establishment a collaborative plan including people with severe asthma and their significant others [39] to optimise sedentary behaviour. Participants in this study, described that doing their planned household chores is one of the main reasons for moving from a sitting to standing position when at home. In line with this finding, doing household chores was reported as one of the most common non-sitting activities in older adults [40]. Additionally, completing household chores is also of importance to people with COPD [26]. Therefore, encouraging people with severe asthma to take on activities within the household may reinforce the benefits that these types of activities can have on breaking up their sedentary behaviour and consequently the associated health benefits.

Misperceptions around sedentary behaviour being the same as physical inactivity are common [41], and observed in this current study. For example, people with severe asthma linked the number of steps performed per day to their level of sedentary behaviour. This indicates a misunderstanding that to reduce sedentary behaviour, requires replacing with walking or purposeful physical activity. This is reflected in intervention studies where a sedentary behaviour change intervention in people with COPD was shown to not reduce time spent being sedentary however increased time spent doing MVPA [42]. The authors attributed this to participants choosing to replace sedentary behaviour with steps, this corroborates that an increase in MVPA does not necessarily translate to a reduction in sedentary time. People with severe asthma spend up to 9 h per day sedentary [4]. It is not feasible, nor expected, that people with severe asthma could replace any significant amount of this time with MVPA. Instead, a more feasible approach would be to replace or break up sedentary behaviour with standing or other LPA including household tasks, as previously discussed [43].

These data provide novel insights from the perspective of people with severe asthma on their perception of sedentary behaviour. To ensure the validity of the data, this study includes a purposeful sample size of people with confirmed severe asthma, providing a larger dataset than previous similar qualitative studies [31, 37, 44]. We acknowledge that the generalisability of our findings may be limited. Participants were recruited from one geographical location and were mostly not working or were retired. Therefore, future studies should incorporate people with a mix of demographic characteristics such as ethnicity and age. It is important to acknowledge that this study is a secondary analysis of interviews, however, the findings add new strength to developing knowledge about sedentary behaviour in people with severe asthma – an area that has not been previously explored in depth. Previous authors have emphasised the value of conducting secondary analysis to explore sensitive issues within an elusive and hard-to-reach population [45]. Future qualitative research that is specifically designed to investigate sedentary behaviour in people with severe asthma is warranted to build upon these initial findings. Additionally, we implemented various strategies to ensure the quality of our secondary analysis [46, 47], including the involvement of a research team member (EM) with fresh perspectives who was not involved in the primary data analysis to also code 30% of the interviews to assess for agreement. We also maintained audit trails and engaged in peer debriefing.

This study provided key elements in understanding the perception of sedentary behaviour in people with severe asthma. Our findings show that future interventions should (1) target periods of long sitting in the afternoon, particularly during TV-viewing time (2) manage asthma and comorbidity symptoms known to enable sedentary behaviour (3) establish a plan to be less sedentary with social peers and promote completing household tasks and (4) provide education on the difference between sedentary and physical inactivity. Future sedentary behaviour intervention targeted at people with severe asthma should incorporate these findings to develop programme theory.

## Electronic supplementary material

Below is the link to the electronic supplementary material.

Supplementary Material 1

## Data Availability

All data generated or analysed during this study are included in this published article.
