# Supplementary material for: Perceptions of sedentary behaviour in people with severe asthma: a qualitative study
Source: BMC Public Health. 2024 Oct 30;24:3011. doi: 10.1186/s12889-024-20446-4 (PMC11526650; doi:10.1186/s12889-024-20446-4)
Supplement: Supplementary file 1 — Supplementary Material 1 [file 12889_2024_20446_MOESM1_ESM.docx]

Journal: BMC Public Health

Supplementary material - Perceptions of sedentary behaviour in people with severe asthma: A qualitative study

Paola D Urroz Guerrero ^1,2,^, Peter G Gibson ^1,2,3^, Hayley Lewthwaite ^1,2^, Eleanor Majellano ^1,2^, Sarah A Hiles ^2,4^, Vanessa M McDonald ^1,2,3^

^1^Centre for Research Excellence in Treatable Traits; College of Health, Medicine, and Wellbeing; University of Newcastle; New Lambton Heights; NSW; Australia.

^2^Asthma and Breathing Program; Hunter Medical Research Institute; New Lambton Heights; NSW; Australia

^3^Department of Respiratory and Sleep Medicine; John Hunter Hospital; New Lambton Heights; NSW; Australia

^4^School of Psychological Sciences; University of Newcastle; Callaghan; NSW; Australia

*Corresponding Author: Professor Vanessa McDonald

Hunter Medical Research Institute, Level 2 West

Locked Bag 1000, New Lambton Heights

NSW, Australia

Vanessa.McDonald@newcastle.edu.au

+61 4042 0146

# Sedentary behaviour focused interview guide questions

Sedentary behaviour is defined as any waking activity characterized by low energy expenditure and a sitting or reclining posture. In general, this means that any time you are sitting or lying down, you are engaging in sedentary behaviour.

- How much of the time do you think you are engaged in sedentary behaviour?
- Tell me what factors influence how much of the time you sit?
- How does your asthma influence the amount of time you spend sitting or lying down?
- Do any other medical problems or symptoms influence the time you spend sitting? What are they and how do they impact?
- What are the reasons that you stop sitting and stand up?
